# Gentamicin Affects the Bioenergetics of Isolated Mitochondria and Collapses the Mitochondrial Membrane Potential in Cochlear Sensory Hair Cells

**DOI:** 10.3389/fncel.2019.00416

**Published:** 2019-09-13

**Authors:** Molly O’Reilly, Luke Young, Nerissa K. Kirkwood, Guy P. Richardson, Corné J. Kros, Anthony L. Moore

**Affiliations:** ^1^Sussex Neuroscience, School of Life Sciences, University of Sussex, Brighton, United Kingdom; ^2^Biochemistry and Biomedicine, School of Life Sciences, University of Sussex, Brighton, United Kingdom

**Keywords:** aminoglycoside, gentamicin, mitochondria, bioenergetics, hair cell, ototoxicity

## Abstract

Aminoglycoside antibiotics are widely prescribed to treat a variety of serious bacterial infections. They are extremely useful clinical tools, but have adverse side effects such as oto- and nephrotoxicity. Once inside a cell they are thought to cause mitochondrial dysfunction, subsequently leading to apoptotic cell death due to an increase in reactive oxygen species (ROS) production. Here we present evidence of a direct effect of gentamicin (the most commonly prescribed aminoglycoside) on the respiratory activities of isolated rat liver and kidney mitochondria. We show that gentamicin stimulates state 4 and inhibits state 3u respiratory rates, thereby reducing the respiratory control ratio (RCR) whilst simultaneously causing a collapse of the mitochondrial membrane potential (MtMP). We propose that gentamicin behaves as an uncoupler of the electron transport chain (ETC) – a hypothesis supported by our evidence that it reduces the production of mitochondrial ROS (MtROS). We also show that gentamicin collapses the MtMP in the sensory hair cells (HCs) of organotypic mouse cochlear cultures.

## Introduction

Aminoglycosides (AGs) are broad-spectrum antibiotics widely prescribed to treat serious bacterial infections such as those leading to septicaemia and meningitis. Although extremely effective clinical agents, they carry the unfortunate risk of adverse side effects such as oto- and nephro- toxicity – damage to hearing and kidney function, respectively, (Mingeot-Leclercq and Tulkens, 1999; Forge and Schacht, 2000; Selimoglu, 2007; Wargo and Edwards, 2014). Nephrotoxicity occurs in approximately 60% of patients treated with AGs (Oliveira et al., 2009). Fortunately this damage is reversible, due to the regenerative abilities of the kidney. However, permanent hearing loss is found in around 20–30% of patients treated with these antibiotics (Rizzi and Hirose, 2007; Schacht et al., 2012), presenting a much more significant clinical concern. The damage to these organs is assumed to be attributable to the selective retention of AGs within these tissue types, with endocytic and non-selective cation channel-mediated routes in kidneys (Nagai and Takano, 2014) and the mechano-electrical transducer (MET) channels of cochlear sensory hair cells (HCs) (Marcotti et al., 2005; Alharazneh et al., 2011) proposed as the main entry pathways.

Once inside a cell AGs are thought to interact with mitochondria, resulting in their dysfunction and the consequent induction of apoptotic cell death cascades. This assumption is based upon several pieces of evidence. Firstly, the remarkable similarity of the structure of mitochondria to that of bacteria (the intended target of AGs) and their proposed bacterial origin indicate that they may represent a secondary target of the antibiotics (Margulis, 1970; Gray et al., 2001; Gray, 2012). Secondly, fluorescence imaging studies showing the co-localization of a fluorescent AG conjugate (gentamicin-Texas Red) with mitochondrial stains such as Mitotracker (Ding et al., 1995; Steyger et al., 2003), suggest that they are trafficked to these organelles once inside the cell. Moreover, point mutations in mitochondrial DNA lead to enhanced susceptibility to AG-induced toxicity (Prezant et al., 1993), again signifying mitochondrial involvement. Lastly, prior to cell death, cells treated with AGs show an increase in the intracellular concentration of reactive oxygen species (ROS) (Clerici et al., 1996; Hirose et al., 1997; Sha and Schacht, 1999) – reactive molecules produced primarily by mitochondria. Taken together these data suggest that mitochondria are a secondary target of the AGs, and that perhaps the resultant mitochondrial dysfunction is the underlying cause of cell death following AG entry and accumulation.

The electron transport chain (ETC) is the driver of oxidative phosphorylation and is the target of a large number of drugs, including several anti-cancer compounds (Rohlena et al., 2011; Kluckova et al., 2013; Olszewska and Szewczyk, 2013). Cisplatin, another nephro- and ototoxicity-inducing compound used to treat several forms of cancer (Skinner et al., 1998; Knight et al., 2005), has been reported to cause mitochondrial dysfunction as a crucial pathogenic event in its induction of nephrotoxicity (Simmons and Humes, 1979). Furthermore, previous studies investigating the effect of gentamicin on renal tissue mitochondria have reported an effect on their state 3 and state 4 respiratory activities, potentially underlying the associated nephrotoxicity (Bendirdjian et al., 1975, 1978; Simmons et al., 1980). However, documentation of the mechanisms underlying the increase in state 4 and decrease in state 3 respiratory activities is lacking. Moreover, the effect of the AGs on cochlear sensory hair cell mitochondria is not fully understood. Studies have reported that AGs trigger the opening of the permeability transition pore, thereby dissipating the mitochondrial membrane potential (MtMP) (Dehne et al., 2002). More recent studies, however, suggest that disruption of the endoplasmic reticulum (ER), or more specifically calcium signaling from the ER to mitochondria, is the earliest pathological event underlying AG-induced ototoxicity (Esterberg et al., 2013, 2014, 2016; Hailey et al., 2017; O’Sullivan et al., 2017).

Here, we present evidence of the direct effect of the AGs on the respiratory function and activity of isolated rat liver and kidney mitochondria. We conducted oxygen consumption assays to investigate the function of the ETC, alongside performing fluorescence-based assessments of the MtMP and mitochondrial ROS (MtROS) production using safranin and Amplex Red dyes, respectively. MtMPs in the sensory HCs of mouse cochlear cultures were also investigated using Rhodamine-123.

Our data suggest that gentamicin, the most commonly prescribed aminoglycoside (Eltahawy and Bahnassy, 1996; Gonzalez, and Spencer, 1998; Xie et al., 2011), stimulates state 4 (non-phosphorylating respiration) and inhibits state 3u (uncoupled) respiratory rates, thereby reducing the respiratory control ratio (RCR) of isolated mitochondria. Our evidence also suggests that gentamicin does not result in MtROS production in isolated mitochondria, but rather reduces its generation. Lastly, we highlight that the documented effects observed in whole cell systems also occur in our isolated mitochondrial assays, suggesting that mitochondria can initiate the effects induced by AGs independent of any input from the ER.

## Materials and Methods

### Isolation of Rat Liver and Kidney Mitochondria

Sprague-Dawley rats of either sex were killed by two methods depending on their weight, according to Home Office (United Kingdom) guidelines. If weighing less than 150 g, cervical dislocation was used whereas if the weight was over 150 g, the rat was killed by exposure to slowly rising concentrations of CO_2_ over a period of 10 min. No difference in mitochondrial function was detected between these two methods, both in terms of respiratory rates and also the response to gentamicin exposure. 25 rats (16 males and 9 females) were used for the experiments presented herein. All rats were within the age range of 1–3 months. Age and sex differences were not investigated. However, comparisons were made regarding the effects of gentamicin on tissue from each animal, so any unequal distribution of animal age/sex would not be a confounding, extraneous variable to the effects that were detected.

The liver or kidney was dissected and transferred to ice cold, 18.2 MΩ resistivity water for approximately 1 min before being placed in buffer solution containing 1 mM EGTA, 30 mM MOPS, 250 mM sucrose, 3.5 mM L-cysteine and 0.1% BSA, pH adjusted to 7.6 using NaOH. The tissue was homogenized with 10 passes in a loose-fitting, followed by 10 passes in a tight-fitting, homogenizer. After filtering through muslin the homogenate underwent differential centrifugation at 4°C – initially at 1000 *g* (after which the supernatant was kept and pellet discarded) and then twice at 10,000 *g*, each for 10 min. After each centrifugation the supernatant was discarded, and the mitochondrial pellet was re-suspended in a small aliquot of buffer solution. Isolated mitochondria were kept on ice before being transferred to the oxygen electrode chambers for the initiation of experimentation.

Mitochondrial protein content was estimated using the Bradford method (He, 2011), with Bio-Rad protein assay dye.

### Measurement of Mitochondrial Respiration

Oxygen consumption rates of isolated mitochondria were measured using an Oroboros Oxygraph-2K oxygen electrode. Assays were performed in rat media containing 200 mM sucrose, 25 mM KCl, 11 mM MgCl_2_, 5 mM KH_2_PO_4_, 5 mM MOPS, pH adjusted to 7.4 using NaOH. Approximately 600 μg of crudely isolated mitochondrial sample was added to the chamber containing 2 ml assay media and allowed to equilibrate for 10 min. The reaction was then initiated with both 5 mM pyruvate and 2 mM malate for complex I-, or 10 mM succinate in the presence of 0.5 μM rotenone for complex II-dependent respiration. Uncoupled respiration was achieved with the addition of 0.5–2 μM CCCP, and complex IV respiration was investigated by addition of 8 mM ascorbate and 4 mM TMPD. All experiments were performed at 32°C.

### Measurement of Respiratory Chain Complexes

All assays were performed via UV-Vis spectroscopy in a 96-well plate format, using a Thermo Scientific Multiskan Go with the Skanit 4.1 software package. Each assay was performed with approximately 100 μg mitochondrial isolate in the same rat media described in measurement of mitochondrial respiration. Prior to the assay, mitochondrial samples were subjected to 2 freeze-thaw cycles to permeabilize the membranes.

#### Complex I

Complex I activity was measured by a protocol adapted from that described in Long et al. (2009). Briefly, 600 μM NADH was added to wells containing ∼ 50 μg mitochondria, followed by 50 μM ubiquinone-2, 2 μM antimycin A and 1 mM KCN in the rat media described above. The rate of NADH oxidation was measured at 350 nm for 10 min.

#### Complex II

Complex II activity was determined by following the reduction of DCPIP at 600 nm over 10 min. The well contained ∼ 50 μg mitochondria in rat media containing 50 μM ubiquinone-2, 74 μM of DCPIP and the reaction was initiated with 100 μM succinate. The protocol was adapted from Medja et al. (2009).

#### Complex III

The activity of complex III was measured by following the rate of reduction of cytochrome C at 550 nm over 5 min. The reaction was initiated by adding 50 μM ubiquinol 2 to wells containing mitochondria in rat media, 64 μM cytochrome C and 1 mM KCN. The protocol was adapted from Medja et al. (2009).

### Measurement of Mitochondrial Membrane Potential and ROS Production

The MtMP was investigated with the use of safranin, a biological stain with an excitation wavelength of 495 nm and an emission wavelength of 587 nm. An initial calibration was performed prior to each experiment by titrating safranin between final concentrations of 0.1 and 2.0 μM in rat media (Krumschnabel et al., 2014). Following calibration, ∼600 μg mitochondrial protein was added and a basal signal (0%) was recorded. A membrane potential was generated upon the addition of 10 mM succinate in the presence of 2.5 μM rotenone, and the sample was subsequently titrated against gentamicin.

Mitochondrial ROS production was measured using Amplex Red, a dye with an excitation wavelength of 563 nm and an emission wavelength of 587 nm. Initial calibrations were performed by first adding 10 μM Amplex Red, 1 U/ml horseradish peroxidase and 5 U/ml superoxide dismutase to rat media for a baseline, with 0.1 μM hydrogen peroxide added to calibrate the signal. Approximately 600 μg mitochondrial protein was added and incubated for 10 min in the presence or absence of 5 mM gentamicin, and respiration was initiated with the addition of 10 mM succinate.

### Measurement of MtMP in Mouse Cochlear Culture Sensory Hair Cells

Rhodamine-123 (1 mg/ml; Vector Laboratories, United States) was diluted 1:200 in an extracellular solution containing: 135 mM NaCl, 5.8 mM KCl, 1.3 mM CaCl_2_, 0.9 mM MgCl_2_, 0.7 mM NaH_2_PO_4_, 5.6 mM D-glucose, 10 mM HEPES-NaOH. MEM (Minimal Essential Medium) amino acids and vitamins, and sodium pyruvate (2 mM) were added from stock concentrates (Fisher Scientific). Cochlear cultures were prepared from postnatal day 2 CD-1 wild type mice of either sex, maintained for 24 h *in vitro*, rinsed 3 times in phosphate buffered saline (PBS) and then incubated in 1 ml of the Rhodamine-123 solution for 15 min at 37°C. Cultures were subsequently rinsed 3 times in PBS and placed in the microscope chamber containing extracellular solution. The sensory HCs were observed with an upright microscope (Olympus) with a 60X water-immersion objective (NA = 0.9). Fluorescence images were obtained using a Visitech VT-Infinity3 confocal system and VoxCell Scan software. Gentamicin (50 mg/ml; Sigma) was added to the recording chamber to a final concentration of 1–20 mM. Fluorescence images were obtained at regular intervals (2–10 min).

The materials used in all the experiments detailed were purchased from Sigma, United Kingdom, unless otherwise stated.

### Statistics

For statistical analyses, *p* < 0.05 was the criterion used for statistical significance. Multiple comparisons were made using 1-way ANOVA with Dunnett or Tukey *post hoc* tests. Means are quoted and shown in Figures ± SEM. ‘N’ denotes the number of rats used and ‘n’ the number of mitochondrial samples tested. Level of statistical significance is shown in Figures as follows: ^∗^*p* < 0.05; ^∗∗^*p* < 0.01; ^*⁣**^p < 0.001. Dose-response curves were fit using non-linear regression, with n_H_ denoting the Hill Coefficient. All statistical analysis was performed with the Graphpad Prism v7.0 software package.

## Results

### Gentamicin Stimulates State 4 and Inhibits State 3u Respiratory Rates in Isolated Rat Liver Mitochondria

**FIGURE 1:**
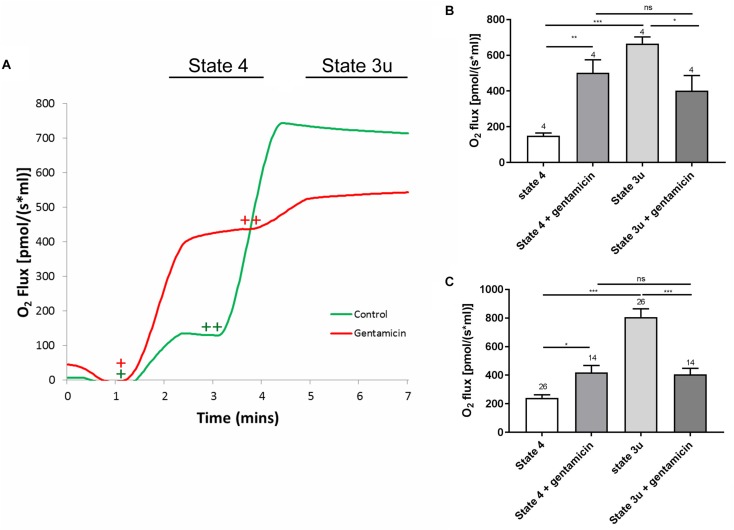
A typical respiratory flux trace for complex II (CII) respiration, alongside quantification of the complex I (CI) and CII responses. **(A)** Approximately 600 μg of mitochondrial protein was pre-incubated in rat medium ±5 mM gentamicin for 10 min. Upon addition of 10 mM succinate (+), a greater oxygen consumption rate can be seen in the sample containing gentamicin (red). Subsequent addition of 1 μM CCCP (++), causes O_2_ flux to rise significantly in the control (green), whereas this effect is reduced in the gentamicin sample. **(B)** Quantification of the CI response, with 5 mM pyruvate, 10 mM glutamate and 2 mM malate addition to stimulate internal NADH generation (*N* = 2, *n* = 4). **(C)** Quantification of the CII response, with 10 mM succinate and 1 μM CCCP used to generate the response (*N* = 5, *n* = 14–26).

The assessment of overall mitochondrial activity was performed by measuring oxygen uptake using an Oroboros Oxygraph, taking advantage of the dual chamber set-up for running control experiments simultaneously. Mitochondrial samples were equilibrated in reaction medium with or without 5 mM gentamicin for 10 min prior to the addition of 10 mM succinate, in the presence of 2.5 μM rotenone to ensure that nicotinamide adenine dinucleotide (NADH) generated within the tricarboxylic acid (TCA) cycle did not become a factor contributing to oxygen flux. Upon addition of the substrate, oxygen consumption increased to 240.9 (±22.1) pmol O_2_ s^–1^ ml^–1^ (*N* = 5, *n* = 26) within the control samples and 419.6 (±48.5) pmol O_2_ s^–1^ ml^–1^ (*N* = 5, *n* = 14) in the gentamicin-treated conditions, demonstrating a just significant (*p* = 0.05) stimulation of complex II-based state 4 respiration (Figures 1A,C). Subsequent addition of 1 μM carbonyl cyanide m-chlorophenyl hydrazone (CCCP), to uncouple oxygen consumption rates from oxidative phosphorylation, increased the rate of respiration to 807.2 (±57.4) pmol O_2_ s^–1^ ml^–1^ (*N* = 5, *n* = 26) within the control samples. However, the gentamicin-induced rate remained relatively stable at 406.6 (±40.9) pmol O_2_ s^–1^ ml^–1^ (*N* = 5, *n* = 14) and was not significantly changed relative to the coupled state (Figures 1A,C).

To ensure the effect was not limited to succinate-dependent respiration, the same set of experiments was run using 5 mM pyruvate, 10 mM glutamate and 2 mM malate to stimulate internal NADH, thereby investigating the complex I respiratory pathway. As with the previous set of experiments, the gentamicin-treated mitochondria had a significantly (*p* = 0.0059) higher rate of state 4 respiration in comparison to the control, with the rate increasing from 149.8 (±15.4) pmol O_2_ s^–1^ ml^–1^ (*N* = 2, *n* = 4) in the controls to 502.5 (±72.7) pmol O_2_ s^–1^ ml^–1^ (*N* = 2, *n* = 4) in those treated with gentamicin. Furthermore, respiratory activity in the presence of gentamicin did not increase when treated with CCCP, measuring 402.3 (±85.0) pmol O_2_ s^–1^ ml^–1^ (*N* = 2, *n* = 4), and was not significantly different relative to the coupled rate (Figure 1B).

Furthermore, there was a significant difference in the uncoupled respiratory rates between the control and gentamicin-treated conditions, both for complex I (*p* = 0.0375) and complex II-mediated (*p* < 0.0001) respiration (Figures 1B,C). This implies that gentamicin reduces state 3u respiratory rates, as has previously been reported elsewhere in the literature (Simmons et al., 1980).

### Gentamicin Reduces the Respiratory Control Ratio of Isolated Rat Liver Mitochondria

**FIGURE 2:**
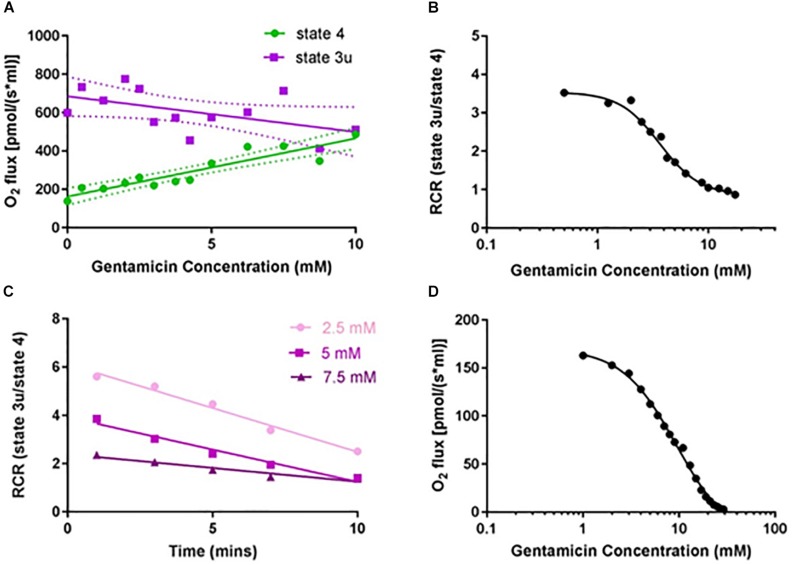
Gentamicin reduces the respiratory control ratio (RCR) of isolated mitochondria in a dose- and time-dependent manner. **(A)** Increase in complex II succinate-induced O_2_ flux (state 4) with increasing gentamicin concentrations, and a simultaneous reduction in the CCCP-induced O_2_ flux (state 3u). Experiments were done in the presence of 2.5 μM rotenone, to inhibit complex I respiration. Lines are fit to a linear regression model, displaying 95% confidence intervals (*N* = 13, *n* = 3–26). **(B)** Transformed data, with RCRs derived by dividing the CCCP-induced O_2_ consumption rates (state 3u) by the succinate-induced O_2_ consumption rates (state 4) (EC_50_ 3.6 mM, n_H_ 2.4). **(C)** Decrease in the RCR with increasing gentamicin incubation times, for three different gentamicin concentrations (2.5, 5, and 7.5 mM) (*N* = 1, *n* = 1). **(D)** Dose-response curve for state 3u respiration with gentamicin at *t* = 10, with an IC_50_ of 9.5 mM (95% CI 8.4–11.0 mM, n_H_ 1.54) (*N* = 1, *n* = 3).

Given that gentamicin stimulates state 4 and causes a concurrent reduction of state 3u respiratory rate (Figure 1), we investigated its effect upon the RCR. Initial dose-response experiments were performed at a fixed 10 min incubation time point, with the data displaying a clear concentration-dependent increase in state 4 respiratory rates (Figure 2A; circles). While the data for uncoupled respiration initially appears erratic (Figure 2A; squares), this is likely due to the non-homogenous nature of the mitochondrial sample making uniform protein addition problematic, and errors becoming more apparent in the faster state 3u rates (*N* = 13, *n* = 26). However, once the data is transformed into RCR values by dividing the uncoupled respiratory rates (state 3u) by the succinate-induced O_2_ consumption rates (state 4), a typical dose-response effect can be observed (Figure 2B).

The extent to which incubation time was a factor was also investigated (Figure 2C). All three gentamicin concentrations tested (2.5, 5, and 7.5 mM) demonstrated a linear reduction in RCR as the length of incubation time increased (*N* = 1, *n* = 1). Dose-response curves (Figure 2D) were generated at *t* = 10 min for state 3u respiration, and an IC_50_ of 9.5 (±2.5) mM (*N* = 1, *n* = 3) was determined.

### Gentamicin Inhibits Complex II and Complex III of the ETC

In order to ascertain if the effects of gentamicin summarized in the above Figures were due to inhibition of the ETC, the overall activities of the complexes were tested in isolation. As with the previous experiments, incubation with 5 mM gentamicin for 10 min prior to the addition of 10 mM succinate and 1 μM CCCP resulted in a reduced state 3u respiratory rate when compared to the control, but subsequent addition of 8 mM ascorbate and 4 mM N,N,N′,N′-Tetramethyl-p-phenylenediamine dihydrochloride (TMPD) together (to act as electron donors to cytochrome C) restored the oxygen consumption rates to 779 (±99) pmol O_2_ s^–1^ ml^–1^ and 814 (±43) pmol O_2_ s^–1^ ml^–1^ (*N* = 2, *n* = 3) for the control and gentamicin-treated samples, respectively. These rates were not significantly different from each other suggesting that the antibiotic had no direct effect upon complex IV (data not shown).

In order to investigate the effects of gentamicin on complexes I, II and III, a spectrophotometric technique was used to measure the activity of individual complexes. When the activity of complex I was measured in the presence of 2 μM Antimycin A and 50 μM ubiquinone-2 as the electron acceptor, to ensure complete isolation of complex I, a discernible reduction in NADH oxidation rate was not detectable up to a gentamicin concentration of 40 mM (data not shown).

**FIGURE 3:**
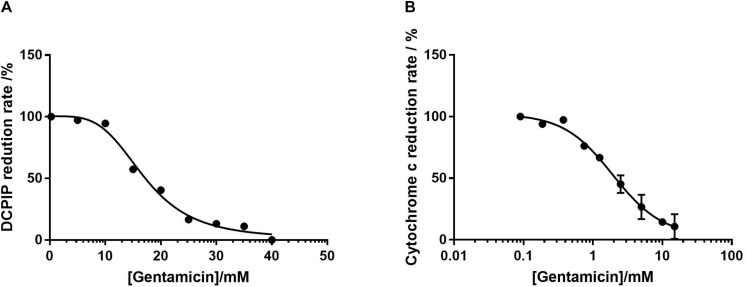
Dose-response curves of isolated mitochondrial protein as a function of gentamicin concentration. **(A)** Inhibition of complex II in isolation, determined by the decrease in rate of DCPIP reduction at 600 nm, giving an IC_50_ of 17 mM (95% CI 14.7–21.6 mM, n_H_ 3.8) for gentamicin. **(B)** Inhibition of complex III, determined by the decrease in cytochrome C reduction at 550 nm rate using ubiquinol-2 as a substrate. The IC_50_ of gentamicin was determined to be 1.94 mM (95% CI 0.7–1.7 mM, n_H_ 1.2). For both panels *N* = 3, *n* = 3.

Complex II activity was determined by following the reduction of DCPIP at 600 nm in the presence of 2 μM Antimycin A and 50 μM ubiquinone-2. Given the previously demonstrated time-dependent nature of inhibition, the mitochondria were incubated with gentamicin for 10 min prior to the addition of succinate. Figure 3A shows that the reduction rate of DCPIP reduces in response to gentamicin in a dose-dependent manner with an IC_50_ of 17 mM, indicating an inhibition of electron transfer from complex II.

The respiratory rate of complex III was measured by following the rate of cytochrome C reduction using 50 μM ubiquinol 2 in the presence of 1 mM cyanide (KCN) to prevent electron transfer to complex IV. As with the complex II assays, Figure 3B shows that gentamicin provides a clear dose-dependent reduction in the rate of electron transfer, with an IC_50_ value of 1.94 mM. Given that the previous assays with intact mitochondria demonstrated a reduction in state 3u respiration rates when pyruvate, glutamate and malate (complex I) and succinate (complex II) were used as substrates, it is highly likely that this is due to the inhibition of complex III.

### Gentamicin Depolarizes the Mitochondrial Membrane Potential

**FIGURE 4:**
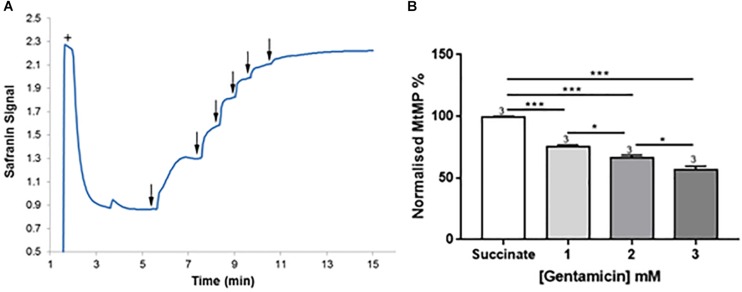
Gentamicin depolarizes the mitochondrial membrane potential (MtMP) in a concentration-dependent manner. **(A)** Succinate is added to the chamber to initiate respiration (+). This generates a proton gradient and consequent polarization of the mitochondrial membrane, shown by the quenching of the signal. When gentamicin is added to the chamber in 2.5 mM increments (↓) up to a concentration of 15 mM, there is a rapid, direct increase in safranin signal, suggesting that gentamicin is dissipating the proton gradient and depolarizing the MtMP. **(B)** Quantification of the results, with data normalized between addition of succinate and safranin (100 and 0%, respectively). A significant shift in safranin signal relative to succinate only is evident at gentamicin concentrations ≥1 mM (*N* = 3, *n* = 3).

Given the apparent effect of gentamicin on the mitochondrial RCR, we investigated its effect on the MtMP using safranin as an indicator of membrane potential (Åkerman and Wikström, 1976; Krumschnabel et al., 2014). Initial addition of safranin (2 μM) to the mitochondrial preparation generates the fluorescent signal. Subsequent addition of succinate (Figure 4A+) in the presence of 2.5 μM rotenone initiates respiration and a proton gradient is formed, with the safranin signal decreasing due to its accumulation within the mitochondrial matrix. Upon addition of 2.5 mM gentamicin (Figure 4A↓) the MtMP gradually depolarizes, with successive 2.5 mM additions (Figure 4A↓) causing further depolarization.

Figure 4B shows quantification of the results. A significant shift in safranin signal relative to succinate only is evident at gentamicin concentrations ≥1 mM (*p* = 0.009) (*N* = 3, *n* = 3). As shown in Figure 4A, complete dissipation of the gradient is observed with successive additions of gentamicin up to a final concentration of 15 mM. However, if lower concentrations of gentamicin (≥1 mM) are added and incubated in the chamber then a progressive depolarization of the MtMP is observed over time (data not shown), suggesting that gentamicin must first cross the outer mitochondrial membrane (OMM) in order to exert its effect on the respiratory proteins embedded within the inner mitochondrial membrane (IMM).

### Gentamicin Reduces Mitochondrial ROS Production With Succinate as the Substrate

**FIGURE 5:**
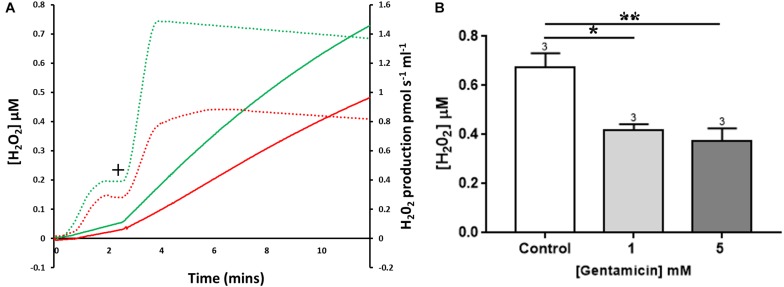
The effect of gentamicin on mitochondrial H_2_O_2_ (ROS) production. **(A)** H_2_O_2_ levels detected over a period of 10 min, with the rates of production and overall concentrations plotted. Solid lines display Amplex signal (H_2_O_2_ concentration), dotted lines display rates of H_2_O_2_ production. Green is control sample, red is pre-treated with gentamicin. Following succinate addition (10 mM) (+), the formation of H_2_O_2_ is reduced in samples that were pre-incubated for 10 min with 5 mM gentamicin. **(B)** Shows quantification of the increase in cumulative H_2_O_2_ over a 10 min period, with final concentrations plotted (*N* = 3, *n* = 3). Gentamicin reduces endogenous ROS production.

Mitochondrial ROS production can be measured using Amplex Red, a dye that fluoresces dependent on hydrogen peroxide (H_2_O_2_) levels. Endogenous H_2_O_2_ production by isolated rat liver mitochondria was measured following the addition of succinate (Figure 5A+). Pre-incubation with 5 mM gentamicin for 10 min caused a reduction in the amount of H_2_O_2_ produced over a subsequent 10-min time period (Figure 5A).

Quantification of the increase in H_2_O_2_ concentrations over time is shown in Figure 5B, with final H_2_O_2_ concentrations generated after 10 min plotted. When compared to the control, there was a significant reduction in the amount of ROS generated when mitochondria were incubated with 1 (*p* = 0.0167) and 5 (*p* = 0.0080) mM gentamicin (*N* = 3, *n* = 3).

### Gentamicin Causes State 4 Stimulation and State 3u Inhibition, RCR Reduction, MtMP Depolarization and a Reduction in MtROS Production in Isolated Rat Kidney Mitochondria

Isolated rat liver mitochondria were used for all of the experiments detailed above due to the ease of isolation and the high yield of mitochondria per dissection. However, as this is not an organ that is damaged by AGs, all experiments were repeated with kidney mitochondria to ensure the same effect was seen, as the kidney is susceptible to AG-induced (nephro-) toxicity and thus represents a more clinically relevant mitochondrial subtype. Moreover, differences have been detected in the functioning of the ETC in mitochondria isolated from different organs (Lotz et al., 2014). It was therefore important to establish that similar effects of gentamicin could also be observed in isolated kidney mitochondria.

**FIGURE 6:**
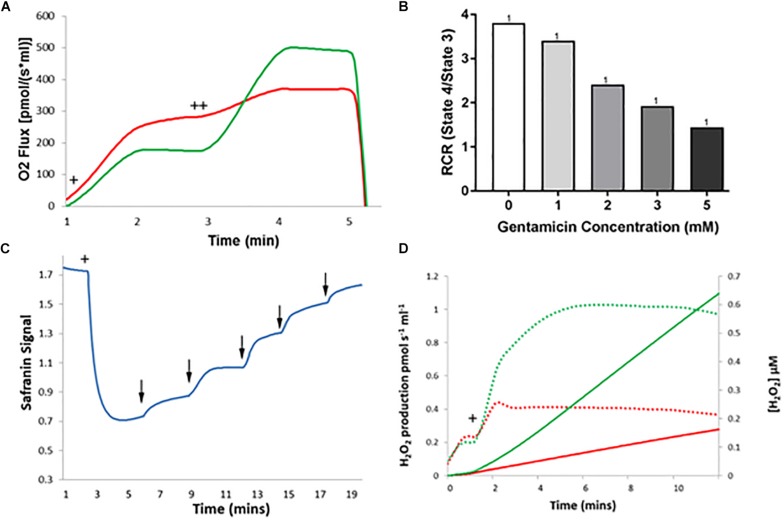
The effect of gentamicin on respiratory parameters in isolated rat kidney mitochondria. **(A)** Succinate addition (+) in the gentamicin condition causes a much larger O_2_ flux response than in the control. Subsequent addition of 1 μM CCCP (++), an uncoupler of the ETC, causes O_2_ flux to rise significantly in the control (green), whereas a greatly reduced rise is seen when pre-incubated with gentamicin (red). **(B)** Quantification of the results shows that gentamicin causes a concentration-dependent decrease in the RCR of isolated kidney mitochondria. **(C)** Succinate is added to the chamber to initiate respiration (+), generating a proton gradient and consequent hyperpolarization of the mitochondrial membrane. When gentamicin is added to the chamber in 1 mM increments (↓) there is a sudden increase in safranin signal, suggesting that gentamicin is dissipating the proton gradient and depolarizing the MtMP. **(D)** Shows H_2_O_2_ levels detected over a period of 10 min, with the rates of production and overall concentrations plotted. Solid lines display concentrations, dashed lines display rates. Green is control, red is gentamicin. Following 10 mM succinate addition (+), the endogenous formation of ROS is inhibited by gentamicin (*N* = 1, *n* = 1).

As shown in Figure 6A, the same stimulation of state 4 and reduction of state 3u respiratory rates were observed, with concurrent reduction in the RCR of kidney mitochondria (Figure 6B). To note, the RCR of kidney mitochondria is much lower than that of liver mitochondria, with an average RCR of 5.28 (±0.83) (*N* = 1, *n* = 4) relative to that of the liver, which was 9.04 (±0.56) (*N* = 1, *n* = 8). This observation is in agreement with a previous report (Lash and Jones, 1993).

Depolarization of the MtMP occurred with serial gentamicin additions (Figure 6C), with a complete collapse of the MtMP at gentamicin concentrations ≥5 mM (*N* = 1, *n* = 1). This is a slight reduction from the 15 mM concentration required to dissipate the MtMP in the liver mitochondria, and we are unable to explain the reason for this at this time. However, given that gentamicin is nephrotoxic, there is potential for mitochondrial morphological differences between kidney and liver samples that have contributed to the increased potency of the compound.

Lastly, gentamicin is shown to reduce ROS production in kidney mitochondria also (Figure 6D), with endogenous ROS levels following succinate addition (Figure 6D+) being greatly reduced in the gentamicin-treated condition relative to the control (*N* = 1, *n* = 1).

### Gentamicin Causes MtMP Depolarization in Sensory Hair Cell Mitochondria

To assess whether the effects observed in isolated mitochondria also occur in intact cell systems, and in particular in the cochlea since it is susceptible to AG-induced ototoxicity, we investigated the effect of gentamicin on the MtMPs in the sensory HCs of mouse cochlear cultures. Previous studies have shown a location-dependent effect of gentamicin on cochlear culture HCs, with basal outer HCs losing their mitochondrial metabolic activity more rapidly than those located in the apical coil (Jensen-Smith et al., 2012).

**FIGURE 7:**
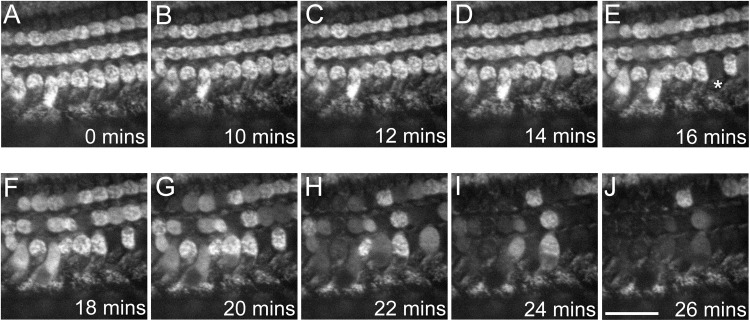
Gentamicin causes a progressive loss of MtMP in mouse cochlear culture HCs after ≥16 min incubation time. **(A–J)** Confocal images show Rhodamine-123 fluorescence in the three rows of outer HCs (OHCs) at differing time points following exposure to 5 mM gentamicin. Loss of fluorescence begins at 16 min post-exposure, with complete loss evident in one OHC (^∗^). The decrease in fluorescence indicates a collapse of the MtMP. By 26 min post-exposure the majority of OHCs in the region of interest had reduced Rhodamine-123 fluorescence indicating a widespread loss of MtMP. The experiment was performed at room temperature (20–22°C). Scale bar is 25 μm.

Cells were pre-loaded with Rhodamine-123, a fluorescent dye that stains mitochondria based on an active MtMP. If the MtMP is dissipated, the fluorescence signal diminishes (Rahn et al., 1991). Cells were bathed in extracellular solution and gentamicin was added to a final concentration of 1, 5, 10 or 20 mM (*n* = 6 cochlear cultures). Figure 7 shows the HC fluorescence over a period of 26 min in 5 mM gentamicin. Between 0 and 14 min there was no change in the fluorescence detected (Figures 7A–D). By 16 min incubation time, however, the MtMPs in some HCs had dissipated (Figure 7E^∗^). By 26 min, almost all of the HCs in the cochlear region under investigation showed a loss of their MtMPs (Figure 7J). MtMP loss was slower in 1 mM and faster in 10 and 20 mM gentamicin (not shown). This directly correlates with that described above in the isolated mitochondrial assay systems, adding further confirmation that loss of the MtMP may be the process underlying AG-induced ototoxicity.

## Discussion

The AGs have long been assumed to target mitochondria once inside the cells of the tissue types that are susceptible to AG-induced toxicity, including renal cells and the sensory HCs of the cochlea. This has largely been based upon indirect lines of evidence showing co-localization of fluorescent mitochondrial dyes with fluorescently conjugated AGs (Ding et al., 1995; Steyger et al., 2003), increased susceptibility to AG damage due to mitochondrial DNA mutations (Prezant et al., 1993), and inferences made on the basis of ROS production (Clerici et al., 1996; Hirose et al., 1997; Sha and Schacht, 1999). Other lines of evidence have also shown a direct effect of the AGs on the respiratory activities of mitochondria and on the permeability of their membranes. Here, we present an investigation of each of these effects, developing a mechanistic hypothesis of how AGs induce mitochondrial dysfunction.

Our initial investigation of isolated mitochondria indicated that gentamicin causes an increase in state 4 respiration rates independent of the initiating substrate, while also causing a consistent reduction in the uncoupled rates, confirming studies that have been undertaken by others previously (Weinberg and Humes, 1980). Investigation of the isolated ETC proteins indicates that inhibition of the *cytochrome bc_1_* complex is the primary cause for reduction in state 3u, which correlates well with the reduction in uncoupled respiration rates when either succinate or pyruvate, glutamate and malate were used as substrates during the multi-complex assays, as complex III is the convergence point for both complex I and complex II pathways. While *in vivo* work has previously shown that mitochondria isolated from gentamicin-treated rats have reduced oxidative phosphorylation turnover (Sahu et al., 2014; Abuelezz et al., 2016; Felix et al., 2017), these do not confirm that the aminoglycoside affects the complexes directly. Gentamicin is known to inhibit mitochondrial protein synthesis via interaction with mitochondrial ribosomes (Hobbie et al., 2008), and as such reduced respiratory capacity could be precipitated by misfolded or reduced levels of ETC proteins. This would explain the discrepancies between our work demonstrating a lack of inhibition at complex I at these concentrations, and others who have shown a reduction in activity of complex I in mitochondria isolated from gentamicin treated rats (Abuelezz et al., 2016).

It should also be noted the IC_50_ was higher in the fully intact mitochondrial samples than the permeabilized samples used in the isolated enzyme assays, which would suggest the OMM is a significant barrier to gentamicin entry. This is likely to be the cause of the time-dependence seen in Figure 2C, as the internal matrix concentration of the compound can equilibrate over time with the external solution.

While the concentrations used within this study appear higher than would typically be pharmacologically relevant, it has been demonstrated previously that there is a significant uptake of gentamicin into renal cortical tissue relative to the blood plasma (up to a concentration of approximately 5 mM) (Simmons et al., 1980). Given that we have demonstrated that the effects are consistent in both liver and kidney mitochondria, it is likely that the primary cause for differences seen in organ pathologies is linked to gentamicin uptake into cells rather than organelle-specific differences in mitochondrial function. AGs can readily enter the sensory HCs of the inner ear specifically, through their MET channels, and have been shown to accumulate at a very high rate. Dihydrostreptomycin, a semisynthetic AG, when at an extracellular concentration of 1 μM, has been estimated to reach an intracellular concentration of 1 μM within 80 s in OHCs (Marcotti et al., 2005), corresponding to 1 mM in 22 h and highlighting the rapid entry and accumulation rates of the AGs into sensory HCs.

When taken in isolation, it is expected that inhibition of complex III would lead to an increase in ROS production, as has previously been shown with Antimycin A (Chen et al., 2003). However, our results have shown that there is in fact a decrease in ROS production in isolated mitochondria, indicating that the increase in ROS levels seen in other studies (Clerici et al., 1996; Hirose et al., 1997; Sha and Schacht, 1999) is not likely mitochondrial in origin, and more likely to be generated by the gentamicin-iron complexes that have been proposed by others (Lesniak et al., 2005). Differences between our protocol and that used by Walker (Walker and Shah, 1988) may also account for the discrepancies between our results and the aforementioned study, as the lack of mitochondrial turnover while the gentamicin is incubating within this study may contribute to the lack of excess peroxide generation. If the mitochondria are already uncoupled prior to respiratory activation then there will not be a high membrane potential, which will reduce RET through complex I, one of the major sources of mtROS production. Other studies (Walker and Shah, 1988; Yang et al., 1995) have also used mitochondria from gentamicin treated rats, where the damage to ETC proteins discussed earlier may cause an increase in the levels of ROS generated.

While it would be tempting to speculate that the inhibition of complex III is the primary cause for dissipation of the MtMP, due to the decrease in proton pumping across the membrane, we do not believe this to be the case. The reduction of the MtMP due to increasing gentamicin concentration (Figure 4) runs concurrent with the increase in state 4 rates (Figure 2A), suggesting there has been a reduction in the proton-motive force thereby allowing the complexes to turn over at a faster rate. It is also a reasonable conclusion that the state 4 rate increase is not the result of activation of any individual ETC complex, as when each complex was tested in isolation, there was no increase in activity seen prior to inhibition in the dose-response curves (Figure 3). However, the reduction in the state 3u rates is also concurrent with the increase in state 4 rates (Figure 2). This would suggest that either there are two separate modes of action for gentamicin, one which is due to inhibition of the individual complexes and one where it dissipates the MtMP, or that the cause of reduction of the MtMP also inhibits the action of the complexes. For the latter to be true, it would suggest that gentamicin directly reduces the integrity of the mitochondrial membrane, first allowing protons to leak across, leading to a reduction in MtMP, followed by reduction of the integrity of the complexes within the membrane.

The mouse cochlear cultures were used in order to confirm our hypothesis that reduction in the MtMP preceded HC death when exposed to gentamicin, and that dissipation of the gradient is not limited to isolated mitochondrial samples. As can be seen in Figure 7 there is progressive dissipation of the MtMP after 15 min of gentamicin exposure, which is substantially faster than the 6 h in 1 mM (Dehne et al., 2002) and 10 h in 3 mM (Servais et al., 2005) time periods that have been described by others Given that the time frame for HC MtMP dissipation is similar to that of isolated mitochondria, it would also appear to confirm that there is rapid uptake of the compound into the cells. As the complete dissipation of the MtMP would prevent ATP generation, HC death would follow once cellular ATP stores have been depleted.

We propose that due to the time- and concentration-dependent nature of both inhibition and uncoupling of the proton-motive force the AGs must first permeate the OMM before exerting their effect on the proteins embedded within the IMM. In order to confirm this postulation, future work should aim to perform electrophysiology on mitochondria and/or mitoplasts (mitochondria that have been stripped of their OMM, leaving the IMM exposed). Gaining electrophysiological recordings of the ionic currents across both membranes would inform us of the possible trafficking of the AGs into mitochondria. Alternatively, the binding of gentamicin to mitochondria has been shown to be persistent and not readily reversible when tested *in vitro* (Kornguth et al., 1980), so perhaps a certain amount must bind before the detrimental effects are observed. Once conclusive, drug development projects could work to design compounds capable of preventing the observed effect of AGs on mitochondria, thereby minimizing the unfortunate side effects associated with these clinically invaluable antibiotics.

## Data Availability

All relevant data are included within the manuscript.

## Ethics Statement

Rats and mice were raised and tissues for experimentation obtained in accordance with Home Office (UK) guidelines.

## Author Contributions

AM, GR, and CK conceived the study. MO, LY, and NK performed the experiments and analyzed the data. All authors contributed to the experimental design and writing the manuscript.

## Conflict of Interest Statement

The authors declare that the research was conducted in the absence of any commercial or financial relationships that could be construed as a potential conflict of interest.
